# Mid-term Effect of Lumbar Sustained Natural Apophyseal Glides in Patients with Non-specific Chronic Low Back Pain: A Randomized Clinical Trial

**DOI:** 10.5152/eurasianjmed.2023.0202

**Published:** 2023-06-01

**Authors:** Şule Şimşek, Nesrin Yağcı, Merve Bergin Korkmaz

**Affiliations:** 1 Department of Therapy and Rehabilitation, Pamukkkale University, Sarayköy Vacational School, Denizli, Turkey; 2 Pamukkale University, School of Physical Therapy and Rehabilitation, Denizli, Turkey; 3 Denizli State Hospital, Denizli, Turkey

**Keywords:** Functional status, kinesiophobia, low back pain, range of motion

## Abstract

**Objective:**

The objective of this study was to compare the standalone and combined mid-term effects of conventional physiotherapy and lumbar sustained natural apophyseal glides on pain, range of motion, fear avoidance belief, and functional status in patients with non-specific chronic low back pain.

**Materials and Methods:**

This randomized clinical study was conducted in a state hospital. Fifty-five patients with non-specific chronic low back pain (mean age: 40.69 ± 6.27 years) were divided into 3 groups. Group I (n = 18) received conventional physiotherapy (electrotherapy and heat application) 5 days a week for 3 weeks, group II (n = 19) received lumbar sustained natural apophyseal glides 3 days a week for 3 weeks. Group III (n = 18) received conventional physiotherapy plus lumbar sustained natural apophyseal glides. Pain (visual analog scale), flexion range of motion (back range of motion II), functional status (Roland–Morris Disability Questionnaire), and fear avoidance belief (Fear Avoidance Belief Questionnaire) were assessed at baseline, third week, and 6-month follow-up.

**Results:**

After 3 weeks of intervention, all outcome measures improved in groups II and III. These improvements remained significant until 6-month follow-up (*P* < .05), except fear avoidance belief (*P* = .06) and flexion range of motion (*P* = .764) scores of group III. Flexion range of motion (*P* = .001), functional status (*P* = .001), and fear avoidance belief (*P* = .03) differed significantly between the 3 groups at 6-month follow-up; post-hoc analysis revealed that flexion range of motion (*P* < .0001), functional status (*P* = .037), and fear avoidance belief (*P* = .002) scores were significantly improved in group II compared to group I.

**Conclusion:**

Compared with conventional physiotherapy, lumbar sustained natural apophyseal glides improved mid-term range of motion, functional status, and fear avoidance belief, but there was no difference in pain. Conventional physiotherapy added to lumbar sustained natural apophyseal glides provided no additional benefit.

**Main Points**

- Activity pain, lumbar flexion range of motion, functional status, and fear avoidance belief were improved with lumbar sustained natural apophyseal glides (SNAGs) in patients with non-specific chronic low back pain (NSCLBP), and these achievements have been maintained at mid-term. However, conventional physiotherapy (CP) improved the functional status and fear avoidance belief only at short term, but these achievements were not maintained at mid-term.
- This made us think that lumbar SNAGs should be preferred instead of CP in patients with NSCLBP.
- The clinical advantages of the lumbar SNAGs are that it can be applied in a short time, it is an active pain-controlled treatment method, and improvements can be achieved immediately after the treatment session.
- The results of this study provided new insights into the effectiveness of manual therapy in patients with NSCLBP.

## Introduction

The point prevalence of low back pain (LBP) is 7.5% in 2017 according to the 2020 Global Burden of Disease data.^1^ Low back pain is the leading cause of activity limitation and absenteeism from work^2^ and results in a huge medical burden and economic cost.^3^ The pain that lasts longer than 7-12 weeks or which recurs intermittently for a long time and the pathoanatomical cause of which cannot be determined is referred as non-specific chronic low back pain (NSCLBP).^4^ Non-specific chronic low back pain represents 90%-95% of LBP cases.^5^ Common symptoms of NSCLBP is pain, decreased range of motion (ROM), functionality, and quality of life.^4^

The main goals of rehabilitation for NSCLBP patients are to control pain, restore function, assure no future functional deficits occur, and preserve employment and productivity.^6^ Conservative management approaches are preferred as a first treatment choice, but the most effective intervention is not clear, and most treatments have little or no effect.^7^ Manual therapy techniques offer moderate- or high-level evidence in the treatment of NSCLBP.^8^ Mulligan concept is essentially an articular technique with neuromuscular consequences. Sustained natural apophyseal glides (SNAGs) is the spine-specific technique of the Mulligan concept.^9^ It is suggested that SNAGs can decrease pain and improve ROM and disability (with moderate evidence) in patients with LBP.^10^

Fear of movement may have a central role in the development of LBP problems and is a strong predictor of self-reported disability in chronic LBP.^11^ Although many studies showed that lumbar SNAGs could provide pain-free ROM, only 1 study has examined fear avoidance belief and reported that a single-session SNAG intervention does not provide any short-term improvement.^12^ The studies in the literature generally focused on the immediate-^13-15^ and short-term^12^ benefits of lumbar SNAGs. Evidence on the mid-term effect of SNAGs is insufficient. Therefore, this study aimed to compare the standalone and combined mid-term effects of conventional physiotherapy (CP) and lumbar SNAGs on pain, ROM, fear avoidance belief, and functional status in patients with NSCLBP.

## Materials and Methods

This randomized, assessor-blind clinical study was conducted in the Denizli State Hospital Outpatient Physical Therapy and Rehabilitation Clinic in Turkey. The study was performed in accordance with the principles of the Declaration of Helsinki and approved by the Pamukkale University Clinical Research and Ethics Committee (Date: November 15, 2016 No: 20). Informed consent was obtained from patients who participated in current study.

The study inclusion criteria were as follows: female gender, being diagnosed with NSCLBP, age between 20 and 50 years, pain duration >3 months, and pain intensity assessed using the visual analog scale (VAS) ranges from 3 to 6. The study exclusion criteria were as follows: low back-related conditions (e.g., spondylolisthesis and spinal stenosis), red flags indicating serious spinal pathology, neurological conditions (e.g., nerve root compromise, neurological signs, disc herniation, and radicular symptoms), rheumatologic or immunologic conditions, psychiatric disorder, cancer, had previous surgery related to the back, pregnancy, other current treatment, and participant’s prior experience with a given treatment.

**Figure 1.**
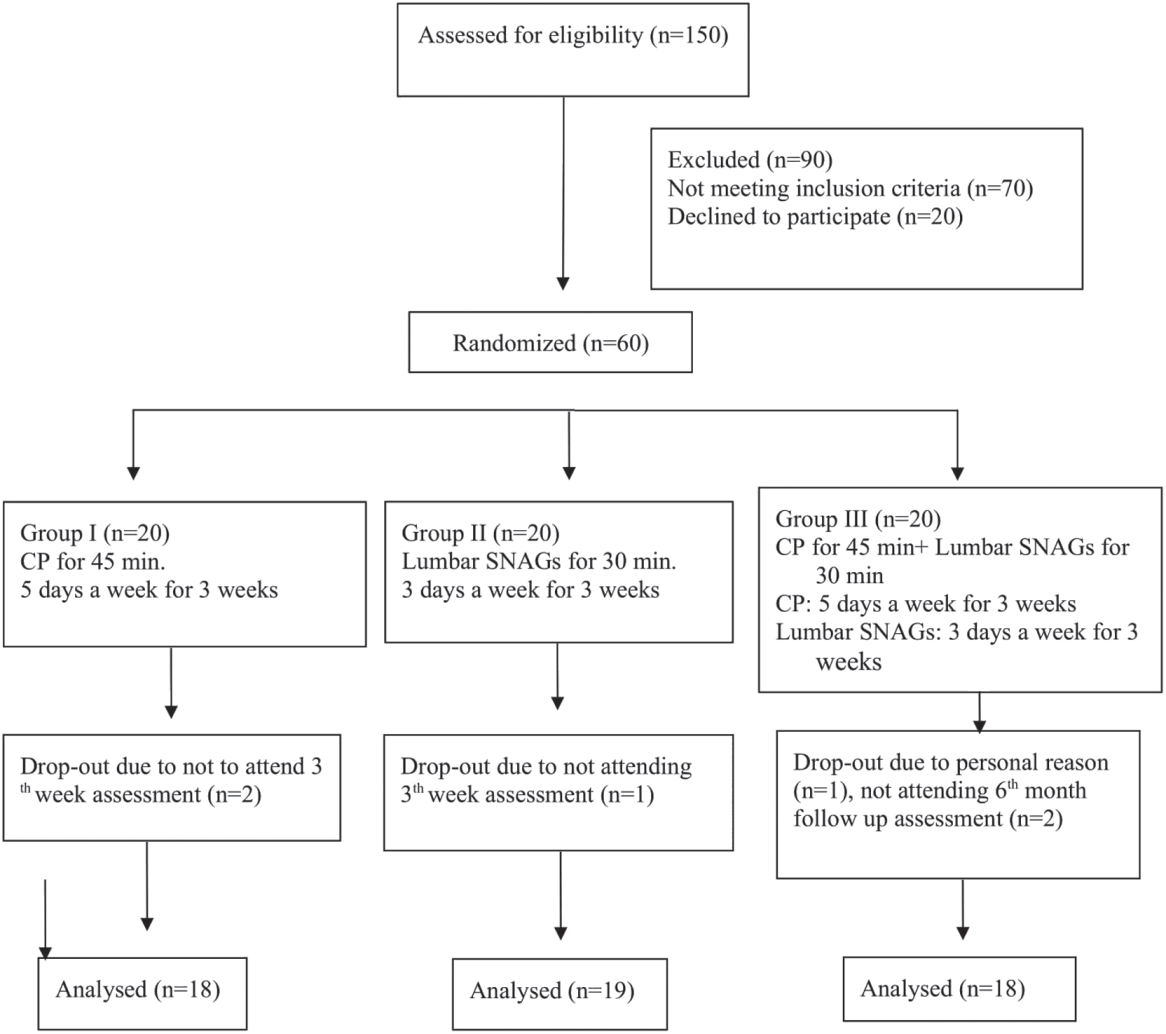
Flowchart of the study sample.

One hundred fifty patients were assessed for eligibility. Twenty-eight participants were excluded from the study because they did not meet the inclusion criteria (n = 20) and did not want to participate in the study (n = 8). Finally, 60 patients were randomly divided into 3 groups (20 patients in each group) using the closed envelope method (Figure 1).

### Group I

Patients assigned to group I received CP for 5 days a week for 3 weeks. Conventional physiotherapy consists of hot pack (20 minutes), therapeutic ultrasound (frequency 1 MHz, intensity 1.5 w/cm^2^, and duration 5 minutes), and transcutaneous electrical nerve stimulation (frequency 50 Hz with <150 microseconds pulse duration and current set in accordance with participant’s sensations for 20 minutes).

### Group II

Patients assigned to group II received lumbar SNAGs 3 times per week for 3 weeks. Lumbar SNAGs were performed by a physiotherapist who is trained in Brian Mulligan’s concepts of Mobilization With Movement (certificated by the Mulligan Concept Teachers Association). The physiotherapist has more than 20 years of clinical experience in the treatment of musculoskeletal conditions. Before applying SNAGs, the patients were evaluated to determine the painful or restricted lumbar segment. The Mulligan concepts lumbar extension SNAGs in prone, SNAGs in lion position, and lumbar flexion SNAGs in sitting techniques were performed. The techniques were applied in 3 sets of 10 repetitions with a 60-second rest.

***Lumbar Extension SNAGs in Prone:*** The participants were positioned in prone, and hands were placed close to the ribs at shoulder level. The therapist grasped the patient across the chest and was asked to perform extension. The therapist glided the predetermined painful or restricted facet joint cranially toward the eyeball. After holding this position for 10 seconds, the patient is asked to return to the initial position while the therapist maintained gliding.^16^

***SNAGs in Lion Position***: In the quadruped position, while patients were instructed to sit between the heels without changing the hand position, the therapist glided the predetermined painful or restricted segment in the lumbar spine. This position is held for 10 seconds and then the participant is asked to return to starting position while the therapist maintained mobilization. Holding for 10 seconds, the participant is asked to return to the initial position, while the therapist maintained the mobilization.^16^

***Lumbar Flexion SNAGs in Sitting***: The participant was asked to sit on the edge of the table, and stool was placed under the feet. The mobilization belt was secured around the patient’s pelvis and around the therapist’s gluteal folds. The therapist glided the predetermined painful or restricted facet joint by pushing it toward the eyeball. The patient was asked to perform the flexion movement and hold for a few seconds and return to the initial position while the therapist maintained gliding.^16^

### Group III

Patients assigned to group III received CP for 5 days a week plus lumbar SNAGs for 3 days a week (first, third, and fifth days of every week) for 3 weeks.

The demographic data (age and body mass index) of the participants were recorded. Pain intensity, ROM, functional status, and fear avoidance belief were assessed. Outcomes were measured at baseline, at the third week (after the intervention), and at 6-month follow-up.

Visual analog scale was used to assess lumbar pain intensity during trunk flexion. Back Range of Motion II (BROM II) was used to assess lumbar flexion ROM. The device was fixed with Velcro at the symphysis pubis level of the patients, and the value on the unit was recorded. The participant was asked to touch the floor with his fingertip, and the value on the unit was recorded again. The difference between the 2 recordings constituted the flexion ROM.

The Roland–Morris Disability Questionnaire (RMDQ) was used to assess physical function. It consists of 24 items regarding activities of daily living that may be affected by back pain. Total score ranges from 0 to 24 points, with a higher score representing extremely severe disability.^17^

Fear Avoidance Belief Questionnaire (FABQ) was used to assess fear avoidance belief. Fear Avoidance Belief Questionnaire is a commonly used condition-specific health status measure for the assessment of fear of movement related to LBP. The score ranges from 0 to 96 points, with a higher score indicating great fear avoidance belief.^18,19^

### Statistical Analysis

The sample size was estimated based on the primary endpoints, which were defined as the immediate effect of the intervention on the VAS and RMDQ scores. The overall effect size of the reference study was large with an index for RMDQ (*d* = 2.34).^13^ Therefore, we included a 3-group comparison with a large effect size (*f* = 0.44). A 3-group comparison with a large size (*f* = 0.44) was included in the study. Accordingly, when at least 54 people (at least 18 for each group) were included in the study that would result in 80% power with a 95% CI.

Obtained data were analyzed using Statistical Package of Social Sciences Statistics (Version 21; IBM, Armonk, NY, USA). Continuous variables were presented as mean ± SD, and maximum, minimum, and categorical variable values were presented as absolute frequency and percentages. The conformity of continuous variables with normal distribution was evaluated using the Shapiro–Wilk test. Paired samples *t*-test for parametric test assumptions and Wilcoxon signed-rank test (baseline to 3-week change in RMDQ scores of group II was not distributed normally) for non-parametric test assumptions were used for pairwise comparisons of within-group change scores. One-way ANOVA (post-hoc Tukey test) (baseline values of VAS and ROM, at third week value of FABQ and ROM, at 6-month value of RMDQ and ROM, baseline to 3-week change scores of VAS, and baseline to 6-month value of RMDQ were distributed normally) for parametric test assumptions and independent samples Kruskal–Wallis test (post-hoc Mann–Whitney *U* test with Bonferroni correction) for non-parametric test assumptions were used for intergroup difference among groups. Statistical significance was set at *P* < .05.

## Results

A total of 60 patients were enrolled in this study. Two patients from group I, 1 patient from group II, and 2 patients from group III were excluded because of their unwillingness to come to the follow-up assessment, and so the final study sample consisted of 55 patients (Figure 1). Group I and group III consisted of 18 patients and group II consisted of 19 patients. The descriptive data of the participants are shown in Table 1.

### Activity Pain

After 3 weeks of intervention, activity pain significantly decreased in group II (*P* ≤ .0001) and group III (*P* ≤ .0001), demonstrating 3.6 and 3.2 point improvements, respectively. However, the within-group scores were not significantly different in group I (*P* = .206). The between-group assessment demonstrated no significant differences at baseline (*P* = .267) but a significant difference at the third week (*P* < .0001). Mean change scores of activity pain differed significantly between the 3 groups at the third week (*P* < .0001); post-hoc analysis revealed that activity pain in group II (*P* < .0001) and group III (*P* = .001) was significantly improved compared to group I. Decreases in activity pain from baseline remained significant in group II (*P* = .006) and group III (*P* = .003) for 6 months. The within-group score of activity pain was statistically significant in group I at 6 months (*P* = .022). The between-group scores (*P* = .119) and mean change scores (*P* = .077) demonstrated no significant differences at 6-month follow-up (Table 2).

### Flexion Range of Motion

After 3 weeks of intervention, flexion ROM significantly increased in both group II and group III (*P* < .05), with an average of 6.6 and 2.9 cm, respectively. However, the within-group scores were not significantly different in group I (*P* = .597). The between-group assessment demonstrated no significant differences at baseline (*P* = .162) and at 6 months (*P* = .710) but a significant difference at the third week (*P* = .019). The mean change scores of flexion ROM significantly differed among the 3 groups at the third week (*P* < .0001) and at 6-month follow-up (*P* = .001); post-hoc analysis revealed that flexion ROM in group II (*P* < .0001) was significantly increased compared to group I (Table 2).

### Functional Status

After 3 weeks of intervention, functional status significantly increased in all 3 groups (*P* < .05), demonstrating 1.6, 4.2, and 5.1 point improvements in group I, group II, and group III, respectively. However, the within-group scores were not significantly different in group I at 6-month follow-up (*P* = .886). The between-group assessment demonstrated no significant differences at baseline (*P* = .056) but a significant difference at third week and 6-month follow-up (*P* < .0001). The mean change scores of the RMDQ at the third week (*P* = .004) and 6-month follow-up (*P* = .01) were significantly different among all 3 groups; post-hoc analysis revealed that functional status improved significantly in group III than in the group I at the third week (*P* = .003) and in group II (*P* = .037) and group III (*P* = .014) compared to group I at 6-month follow-up (Table 2).

### Fear Avoidance Belief

After 3 weeks of intervention, fear avoidance belief significantly decreased in all 3 groups (*P* < .05), demonstrating 1.8, 4.6, and 6.2 point improvements in group I, group II, and group III, respectively. The between-group assessment demonstrated statistically significant differences at baseline (*P* = .026) and at the third week (*P* = .031). The mean change scores of FABQ significantly differed among the 3 groups at the third week (*P* = .004); post-hoc analysis revealed that fear avoidance belief in group II was significantly improved compared to group I (*P* = .003). The within-group score was statistically significant in group II at 6-month follow-up (*P* ≤ .0001). The between-group assessment demonstrated statistically significant differences at 6-month follow-up (*P* < .0001). The mean change scores of FABQ significantly differed among the 3 groups at 6-month follow-up (*P* = .003); post-hoc analysis revealed that fear avoidance belief was significantly improved in group II compared to group I (Table 2).

## Discussion

The aim of this study was to compare the standalone and combined mid-term effects of CP and lumbar SNAGs on pain, ROM, fear avoidance belief, and functional status in patients with NSCLBP. The results of this study showed that pain, ROM, functional status, and fear avoidance belief improved after 3 weeks of lumbar SNAGs, and these achievements were maintained up to the mid-term. In contrast, the achieved improvements in functional status and fear avoidance belief with CP did not maintain up to the mid-term. The mid-term improvements in ROM, functional status, and fear avoidance belief with lumbar SNAGs were significantly better than with the CP. Conventional physiotherapy added to lumbar SNAGs provided no additional benefit at any measurement time.

The mechanisms by which Mulligan concept exerts its curative effect in clinical practice still remain mysterious. It has been suggested that the immediate pain relief achieved with SNAGs may be due to non-opioid endogenous pain inhibition pathways or to restoring muscle balance due to correction of positional fault.^20^ Some studies focused on the effect of SNAGs on VAS resting pain score,^13,20^ while others focused on pain activity score.^12,2^ Reduction in pain during flexion was achieved with lumbar SNAGs compared to the control subjects^21^ and sham SNAGs.^12,14^ In the current study, after 3 weeks of intervention, pain during flexion was significantly reduced in the SNAG-administered patients. Patients treated with standalone and combined lumbar SNAGs showed improvement in VAS activity score of 3.6 and 3.2 points, respectively. The minimal clinically important difference (MCID) of VAS pain score in chronic LBP has been reported as 20 mm.^22^ According to the reported MCID score, these improvements may be both considered clinically important and statistically significant after 3 weeks of intervention. At mid-term, pain during trunk flexion was significantly decreased in all intervention groups. While this decrease was 0.8 points in patients who received CP, it was 2.2 and 2.9 points in patients who received standalone and combined SNAGs, respectively. However, the improvement in terms of activity pain achieved with CP intervention is statistically significant but may not be clinically important. Pain during activity has a greater association with decreased physical function and quality of life than pain at rest.^23^ Therefore, we chose to evaluate pain during activity. We think that activity pain is decreased because mobilization is applied in the direction of painful movement. Conventional physiotherapy intervention, which is one of the passive treatment methods, was insufficient to reduce pain during activity. Therefore, adding CP to lumbar SNAGs provided no additional benefit.

Applying SNAGs to mobilize the affected facet joints may release capsular strain, resulting in ROM improvement.^20^ However, studies have focused on the immediate- and short-term effects of SNAGs on ROM^12,14^. Studies have shown that lumbar SNAGs provide pain-free ROM. However, no studies were found examining the mid-term effect of SNAGs. According to the current study results, flexion ROM was significantly increased in the SNAG-administered groups after 3 weeks of intervention, but this improvement was maintained at mid-term only in in patients who were administered standalone lumbar SNAGs. Lumbar SNAGs which was applied in the direction of painful or restricted motion were able to increase ROM in the short term because of provided positive feedback. Thus, patients may be able to repeat painful or restricted movements they have experienced before without fear, and these repeated movements may have increased ROM. The improvement in ROM may not have been maintained in the mid-term because of the high baseline FABQ score of patients who were administered CP plus lumbar SNAGs intervention. In addition, since the patients were not supported with a self-exercise program after all interventions, the improvements may not have been maintained at mid-term.

In current study, after 3 weeks of intervention, the functional status score was significantly decreased in all intervention groups. While this decrease was 1.6 points in patients who received CP, it was 4.2 and 5.1 points in patients who received standalone and combined SNAGs, respectively. The MCID of functional status RMDQ score has been reported as 3.5 points.^22^ The improvement achieved with CP intervention was statistically significant but may not be clinically important. The improvement in the functional status score was maintained up to mid-term in the SNAG-administered patients. Trunk flexion is known as the most painful movement in patients with LBP. Restriction of trunk flexion may have a major impact on high level of disability in patients with chronic LBP.^23^ In the current study, the achievement of pain-free flexion ROM with lumbar SNAG intervention may lead to an increase in functional status in the SNAG-administered patients.

In the treatment of chronic LBP, it is recommended to avoid long-term inactivity and increase physical activity level gradually.^24^ However, these recommendations might not be followed by the patients who have greater fear avoidance beliefs.^25^ Therefore, it is important to eliminate it. While a previous study had reported that lumbar SNAGs have a short-term favorable effect on fear avoidance belief,^12^ we found that lumbar SNAGs were more effective in reducing fear avoidance belief after 3 weeks of intervention and at mid-term. The mobilization is performed in the direction of previously experienced painful or restricted movement with the SNAGs intervention; therefore, the patient may have gained a positive experience with the painful/restricted movement they had before. And the belief in fear avoidance may have decreased as a result of this. They applied 1 session of SNAGs, whereas we applied 9 sessions. Most probably, this is the reason why we achieved mid-term improvements.

### Limitations

The re-evaluation schedule of primary outcome measures in the current study was at third week and 6-month follow-up. The follow-up period after treatment was relatively long. An interim evaluation during this process could have helped us better interpret the intervention effects. We have chosen the flexion ROM as one of the outcome measures. According to Atya, lumbar flexion cannot be used separately as a collective score or index for disability.^23^ In future studies, rotation and extension ROM and pain during extension and rotation could be included. However, the current study is the first to investigate the standalone effects of the interventions that make up combined intervention and to have mid-term follow-up.

In conclusion, improvements in pain, flexion ROM, functional status, and fear avoidance belief were achieved with lumbar SNAGs. These achievements have been maintained at mid-term. On the other hand, CP reduced the functional status and fear avoidance belief only at short term, but these achievements were not maintained at mid-term. This made us think that lumbar SNAGs should be preferred instead of CP in patients with NSCLBP. The clinical advantages of the lumbar SNAGs are that it can be applied in a short time, it is an active pain-controlled treatment method, and improvements can be achieved immediately after the treatment session. The results of this study provided new information regarding the effectiveness of manual therapy in the treatment of patients with NSCLBP.

## Figures and Tables

**Table 1.** Comparison of the Demographic Variables of Intervention Groups

Variables	Group I	Group II	Group III	*P*
	Mean ± SD (median)	Mean ± SD (median)	Mean ± SD (median)	
Age (years)	39.44 ± 5.08 (40.5)	42 ± 7.51 (42)	40.56 ± 5.97 (42)	.171
BMI (kg/cm^2^)	26.54 ± 4.29 (25.3)	25.68 ± 4.79 (25.3)	26.64 ± 3.57 (25.8)	.753
Employment status	**n (%)**	**n (%)**	**n (%)**	
White-collar jobs	7 (38.9)	13 (68.4)	8 (44.4)	
Blue-collar jobs	11 (61.1)	6 (31.6)	10 (55.6)	

BMI, body mass index.

**Table 2.** All Outcome Measures from Baseline to 6-Month Follow-Up

Variables	Group I	*P*_intragroup _	Group II	*P*_intragroup_	Group III	*P*_intragroup_	*P*_intergroup_
	Mean ± SD (95% CI)		Mean ± SD (95% CI)		Mean ± SD (95% CI)		
VAS—Flexion							
Baseline	5.9 ± 1.6 (5.1-6.7)		5.4 ± 2.3 (4.3-6.5)		6.5 ± 1.7 (5.6-7.3)		.267
3W	5.5 ± 1.8 (4.6-6.3)		1.8 ± 1.6 (1.1-2.6)		3.3 ± 2.7 (2-4.6)		**<.0001**
3W-baseline change	–0.5 ± 1.5 (–0.3 to 1.2)	.206	–3.6 ± 2.1 (–4.6 to –2.6)	**<.0001**	–3.2 ± 2.6 (–4.5 to –1.9)	**<.0001**	**<.0001** ^*,†^
6M	5.1 ± 1.6 (4.3-4.8)		3.2 ± 2.6 (1.9-4.5)		3.6 ± 2.9 (2.1-5.1)		.119
6M-baseline change	–0.8 ± 1.6 (–1.7 to –0.1)	**.022 **	–2.2 ± 3.1 (–3.8 to –0.7)	**.006**	–2.9 ± 3.5 (–4.6 to –1.1)	**.003**	.077
ROM—Flexion							
Baseline	15.8 ± 4.7 (13.5-18.2)		13.3 ± 5.9 (10.7-16.4)		16.9 ± 5.1 (14.3-19.5)		.162
3W	15.2 ± 5.1 (12.7-17.8)		20.21 ± 5.72 (17.5-23)		19.8 ± 6.2 (16.7-22.9)		**.019**
3W-baseline change	–0.6 ± 3.9 (–2.5 to 1.3)	.597	6.6 ± 4.2 (4.6-8.7)	**<.0001**	2.9 ± 5.4 (0.2-5.6)	**.035**	**<.0001** ^*^
6M	13.2 ± 4.3 (11.1-15.3)		18.95 ± 6.9 (15.6-22.3)		17.4 ± 6.3 (14.3-20.5)		.710
6M-baseline change	2.6 ± 4.2 (–4.7 to –0.5)	**.018**	5.4 ± 6.1 (2.5-8.3)	**.001**	0.5 ± 6.9 (–3 to 4)	.764	**.001** ^*^
RMDQ							
Baseline	14.2 ± 2.8 (12.8-15.6)		10.6 ± 4.8 (8.3-12.9)		12.7 ± 3.9 (10.8-14.7)		.056
3W	12.6 ± 4.1 (10.6-14.6)		6.4 ± 3.7 (4.6-8.2)		7.6 ± 3.5 (5.9-9.4)		**<.0001**
3W-baseline change	–1.6 ± 3 (–3.1 to –0.1)	**.043**	–4.2 ± 3.9 (–6.1 to –2.3)	**<.0001**	–5.1 ± 2.9 (–6.5 to –3.7)	**<.0001**	**.004** ^†^
6M	14.1 ± 3.5 (12.3-15.8)		6.8 ± 3.9 (4.9-8.7)		8.3 ± 4.9 (5.9-10.8)		**<.0001**
6M-baseline change	–0.1 ± 3.2 (–1.7 to 1.5)	.886	–3.8 ± 4.7 (–6.1 to –1.5)	**.002**	–4.4 ± 5.1 (–6.9 to –1.9)	**.002**	**.010** ^*^ **^,^** ^†^
FABQ							
Baseline	30.7 ± 5.2 (28.1-33.3)		28.6 ±9.2 (24.2-33)		35.5 ± 8.9 (31.1-39.9)		**.026** ^‡^
3W	28.9 ± 5.8 (26.1-31.8)		24 ± 7.3 (20.5-27.5)		29.3 ± 7.1 (25.8-32.8)		**.031**
3W-baseline change	–1.8 ± 2.5 (–3.1 to –0.5)	**.008**	–4.6 ± 5.6 (–7.3 to –1.9)	**.002 **	–6.2 ± 9.7 (–11 to –1.4)	**.005**	**.004** ^*^
6M	30.4 ± 5.3 (27.8-33)		22.2 ± 8.9 (17.9-26.5)		32.9 ± 8.1 (28.9-37)		**<.0001**
6M-baseline change	–0.3 ± 3.5 (–2.1-1.4)	.655	–6.4 ± 6.4 (–9.4 to –3.3)	**<.0001**	–2.6 ± 9.3 (–7.2 to 2.1)	.06	**.003** ^*^

FABQ, Fear Avoidance Belief Questionnaire; M, months; RMDQ, Roland–Morris Disability Questionnaire; ROM, range of motion; VAS, visual analog scale; W, week. Statistical significance: p<0.05.

^*^CP group vs. SNAG group.

^†^CP group vs. plus group.

^‡^SNAG group vs. plus group.
